# Identification of cytotoxic constituents from Siegesbeckiae Herba and network pharmacology prediction of their anti-pancreatic cancer mechanisms

**DOI:** 10.1038/s41598-025-18358-3

**Published:** 2025-09-26

**Authors:** Kun Zhang, Yi-Ying Zhao, Xi-Wen Duan, Yu-Bin Xu, Yao Yao, Chun-Xue You, Yun-Feng Cui, Yu-Qing Jiang

**Affiliations:** 1 Department of Hepatobiliary and Pancreatic Surgery, Department of Surgery, Tianjin Nankai Hospital, Nankai Clinical School of Medicine, Tianjin Medical University, Tianjin, 300100 China; 2 Graduate school, Tianjin Medical University, Tianjin, 300070 China; 3 Tianjin Key Laboratory of Agricultural Animal Breeding and Healthy Husbandry, College of Animal Science and Veterinary Medicine, Tianjin Agricultural University, Tianjin, 300392 China; 4 College of Biological Science and Technology, Shenyang Agricultural University, Shenyang, 110866 China; 5 Taizhou Central Hospital (Taizhou University Hospital), Taizhou, 31800 China; 6 Teaching and Experiment Center, Liaoning University of Traditional Chinese Medicine, Shenyang, 110847 China

**Keywords:** Siegesbeckiae Herba, Pancreatic cancer, Flavonoids, Cytotoxicity, Network pharmacology, Molecular docking, Biochemistry, Cancer, Chemical biology, Computational biology and bioinformatics, Drug discovery

## Abstract

Pancreatic cancer remains a challenging malignancy with limited treatment options. This study aimed to isolate bioactive compounds from Siegesbeckiae Herba and explore their potential mechanisms against pancreatic cancer through network pharmacology and molecular docking. Twenty compounds, including one new compound, were isolated via chromatographic methods and structurally identified by NMR and mass spectrometry. Cytotoxicity of these compounds was evaluated against the human pancreatic cancer cell line PANC-1 using the CCK-8 assay. Two flavonoid constituents, compound **16** (8, 3’-dihydroxy-3, 7, 4’-trimethoxy-6-O-β-D-glucopyranosyl) and compound **17** (quercetin-3-methyl ether), exhibited cytotoxicity, with compound **16** showing the most potent activity (IC_50_ = 4.48 ± 0.74 µg/mL). Network pharmacology analysis identified 182 potential targets for these compounds, including key targets such as AKT1, PIK3CA, SRC, and HRAS etc. Gene Ontology (GO) and Kyoto Encyclopedia of Genes and Genomes (KEGG) analyses indicated enrichment of critical pathways such as PI3K-Akt, Ras, and HIF-1 signaling, which regulate cell proliferation, migration, inflammation, and apoptosis. Molecular docking confirmed strong interactions between compounds **16** and **17** and key pancreatic cancer targets, yet the two flavonoids occupied distinct active pockets. Notably, compound **16** displayed stronger binding affinities (<-7.8 kcal/mol) toward top-ranked targets including HRAS, PIK3CB, PIK3CA, and HSP90AA1. Our results suggest flavonoids from Siegesbeckiae Herba, especially compound **16**, as promising candidates for pancreatic cancer treatment, warranting further pharmacological investigation.

**Supplementary Information:**

The online version contains supplementary material available at 10.1038/s41598-025-18358-3.

## Introduction

Pancreatic cancer is considered the most lethal human malignancy, with the lowest 5-year relative survival rate among all cancers at just 13% in America and 6.5% in Southeastern China^1,2^. This dismal prognosis is primarily due to resistance to chemotherapeutics and high rates of metastasis^3^. The emergence of drug resistance mandates an urgent need to search for novel sources of therapeutic agents. Traditional Chinese medicine (TCM) is an excellent source of bioactive compounds for the development of chemotherapeutic agents^4^.

Siegesbeckiae Herba, dried aerial parts of *Sigesbeckia orientalis* L., *Sigesbeckia pubescens* Makino or *Sigesbeckia glabrescens* Makino, has been recognised as an important TCM since the Tang Dynasty^5^. Siegesbeckiae Herba, noted for its therapeutic properties in alleviating rheumatism, enhancing joint function, and facilitating detoxification, has been identified as containing multiple bioactive components, including sesquiterpenes, diterpenoids, flavonoids, and others^6^. Siegesbeckiae Herba has demonstrated inhibitory effect on many kinds of tumours. The ethyl acetate and n-butanol extracts of of *S. orientalis* exhibit significant inhibitory effects on the in vitro proliferation of HeLa cells^7^. The essential oil of *S. pubescens* obviously inhibited the proliferation of HepG2 cells in a dose-dependent manner^8^. The aqueous extract of *S. glabrescens* exert anti-proliferative action in human breast carcinoma cells (MDA-MB-231 and MCF-7)^9^. A sesquiterpene lactone from *S. glabrescens* exerted anti-proliferative effects against human pancreatic cancer PANC-1 and AsPC-1 cells^10^. Research on the antitumor effects of Siegesbeckiae Herba has primarily focused on crude extracts, with a lack of studies on individual components.

The present study was designed to isolate and characterize the chemical constituents of Siegesbeckiae Herba and to evaluate their anticancer effects against PANC-1. Twenty compounds were isolated from this herb, including one new compound. Among these compounds, the flavonoid constituents 8,3′-dihydroxy-3,7,4′- trimethoxy-6-O-β-D-glucopyranosylflavone (compound **16**) and quercetin-3-methyl ether (compound **17**) exhibited cytotoxicity against PANC-1 cells, with compound **16** showing the most pronounced effect. However, neither of these compounds has previously been reported for inhibitory effects against pancreatic cancer. Given their low isolation yields, network pharmacology was applied to predict their anti-pancreatic cancer mechanisms. Network pharmacology integrates chemical structure data, target prediction and disease databases to construct a compound-target-disease interaction network and employs pathway enrichment analyses to elucidate natural product mechanisms^11^. This efficient, cost-effective approach comprehensively reveals how active components intervene in disease progression and provides theoretical support for subsequent experimental validation^12^. A total of 182 overlapping targets between the compounds and pancreatic cancer were identified. Protein-protein interaction (PPI) network construction highlighted key targets of these active components, including SRC, AKT1, EGFR, etc. Gene Ontology (GO) and Kyoto Encyclopedia of Genes and Genomes (KEGG) enrichment analyses indicated that these components may act via pathways such as PI3K-Akt signalling, HIF-1 signalling pathway.

In summary, this work reports a new natural product and two flavonoids with anti-pancreatic cancer activity, and provides a mechanistic perspective through a Network Pharmacology. These findings position Siegesbeckiae Herba as a promising source of lead compounds and demonstrate the utility of network pharmacology for guiding subsequent experimental validation.

## Results

### Compounds isolated from Siegesbeckiae Herba

**Fig. 1:**
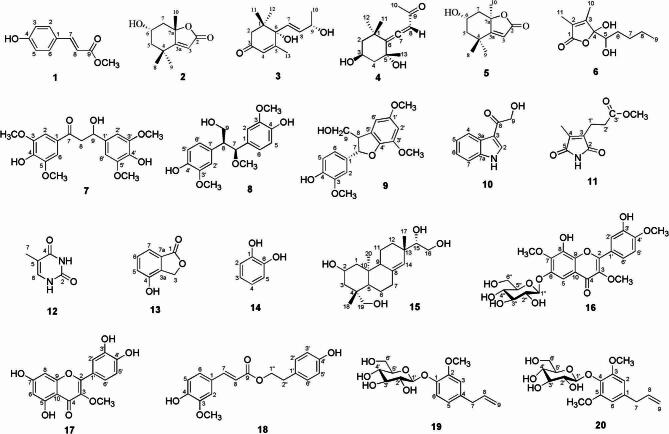
The structures of compounds 1–20.

Twenty compounds were obtained from Siegesbeckiae Herba. Their structures were elucidated by NMR spectroscopy and high-resolution mass spectrometry, as illustrated in Fig. 1. One compound is new, namely 3-hydroxy-1,3-bis-(4-hydroxy- 3,5-dimethoxy-phenyl)-propan-1-one (**7**). The remaining compounds were identified as methyl p-coumarate (**1**), (+)-epiloliolide (**2**), corchoionol C (**3**), lyratol F (**4**), (-)-loliolide (**5**), schumannione (**6**), thero-2,3-bis-(4-hydroxy-3- methoxypheyl)-3-methoxy-propanol (**8**), (7S, 8S)-4-hydroxy-3, 1’, 3’-trimethoxy-4’, 7-epoxy-8, 5’-neolign-9-ol (**9**), 1 H-indole-3-hydroxyacetyl (**10**), methyl hematinate (**11**), thymine (**12**), 4-hydroxy-isobenzofuran-1 (3 H)-one (**13**), pyrocatechol (**14**), kirenol (**15**), 8, 3’-dihydroxy- 3, 7, 4’-trimethoxy-6-O-β-D-glucopyranosyl (**16**), quercetin 3-methyl ether (**17**), p-hydroxyphenethyl trans-ferulate (**18**), 2-methoxy-4-(2-propenyl)phenyl β-D- glucopyranoside (**19**), 4-allyl-2, 6-dimethoxyphenyl glucoside (**20**). Compounds **7**–**9** and **18** are lignans; compound **1**, **19**–**20** are classified as phenylpropanoids; compounds **2**–**6**, **15** are terpenoids; whereas compounds **16** and **17** belong to the flavonoids.

### Structure Elucidation of the new compound

**Fig. 2:**
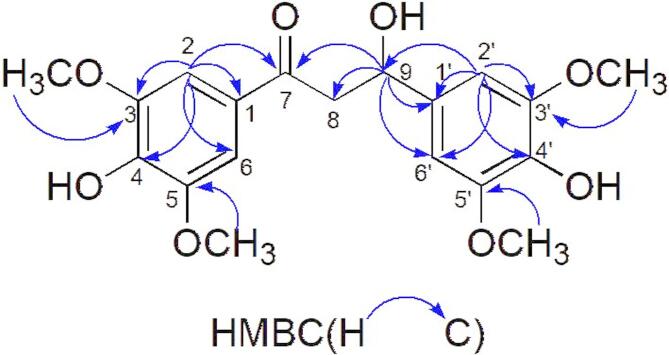
Key HMBC correlations of compound 7.

Compound **7** was obtained as yellow oil that turned pink on spraying with 5% vanillin-H_2_SO_4_. Based on the HR-ESI-MS signal at m/z 379.1390 [M + H]^+^ (calculated for C_19_H_23_O_8_, 379.1393), molecular formula of the compound was assigned as C_19_H_22_O_8_. The ¹³C-NMR spectrum (19 resonances) revealed a carbonyl carbon at *δ*_C_ 198.3 (C-7), oxygenated aromatic carbons at *δ*_C_147.7 (C-3’,5’), 146.7 (C-3,5), *δ*_C_ 140.0 (C-4), and 134.5 (C-4’). Aromatic carbons signals appeared at *δ*_C_ 128.0 (C-1), 127.9 (C-1’), 106.5 (C-2,6), 105.0 (C-2’,6’). The signal at *δ*_C_ 56.5 corresponds to the methoxy carbon (-OCH_3_). In the ¹H-NMR spectrum, two singlets at *δ*_H_ 7.26 (2 H, s, H-2,6) and 6.48 (2 H, s, H-2’,6’) accounted for the aromatic protons, while multiplets at *δ*_H_ 4.60 (1H, m, H-9) and 4.25 (1H, m, H-8) represented the proton signal of a alkoxy group. Four methoxy singlets were observed at *δ*_H_ 3.87 and 3.85 (each 6 H, s, OCH_3_). Key HMBC correlations (Fig. 2) from H-2, 6 to C-1, C-4, C-2,6, and C-3,5, from H-2’,6’ to C-1’, C-4’, C-2’,6’, C-3’,5’, from H-9 to C-8, C-1’, and C-2’,6’, and from the methoxy protons to C-3,5 and C-3’,5’, respectively. On the basis of the above evidence, compound **7** was characterised as 3-hydroxy-1,3-bis(4-hydroxy-3,5-dimethoxyphenyl)propan-1-one. A comprehensive SciFinder search indicated that this substance has not been previously reported. The^1^H- and^13^C-NMR data are summarised in Table 1, and all spectra are provided in the Supplementary Materials.

**Table 1:** ^1^H and ^13^C-NMR data of compound 7 in CDCl_3_.

Position	δ_H_ (ppm)	δ_C_ (ppm)
1		128.0
2,6	7.26 (2 H, s)	106.5
3,5		146.7
4		140.0
7		198.3
8	4.25 (1 H, m) 3.87 (1 H, overlap)	65.4
9	4.60 (1 H, m)	56.5
1’		127.9
2’,6’	6.48 (2 H, s)	105.0
3’,5’		147.7
4’		134.5
3,5-OCH₃	3.87 (6 H, s)	56.5
3’,5’-OCH₃	3.85 (6 H, s)	56.5

### Structure Elucidation of other compounds

Methyl p-coumarate (**1**) was obtained as a yellow oil and developed a pink color upon spraying with 5% vanillin-H_2_SO_4_. ^1^H-NMR (600 MHz, CD_3_OD) *δ* ppm: 7.56 (1H, d, J = 15.8 Hz, H-7), 7.42 (2 H, d, J = 8.5 Hz, H-2,6), 6.79 (2 H, d, J = 8.5 Hz, H-3,5), 6.27 (1H, d, J = 15.8 Hz, H-8), 3.35 (3 H, s, 9-OCH_3_). ^13^C-NMR (150 MHz, CD_3_OD) *δ* ppm: 171.5 (C-9), 161.0 (C-7), 146.2 (C-4), 131.0 (C-1), 127.3 (C-2, 6), 116.8 (C-3, 5), 115.8 (C-8), 49.8 (9-OCH_3_)^13^.

(+)-Epiloliolide (**2**) was obtained as a white powder and developed a yellow coloration upon spraying with 5% vanillin-H_2_SO_4_. ^1^H-NMR (600 MHz, CD_3_OD) *δ* ppm: 5.78 (1H, s, H-3), 4.10 (1H, m, H-6), 2.47 (1H, m, H-7a), 2.01 (1H, m, H-5a), 1.59 (3 H, s, H-8), 1.41 (1H, t, *J* = 11.70 Hz, H-7b), 1.31 (3 H, s, H-9), 1.28 (1H, overlap, H-5b), 1.28 (3 H, s, H-10). ^13^C-NMR (150 MHz, CD_3_OD *δ* ppm: 183.9 (C-3a), 174.0 (C-2), 113.7 (C-3), 88.6 (C-7a), 65.2 (C-6), 50.7 (C-5), 49.4 (C-7), 36.2 (C-4), 30.3 (C-8), 25.8 (C-9), 25.3 (C-10)^14^.

Corchoionol C (**3**) was obtained as a yellow oil and developed a purple coloration upon spraying with 5% vanillin-H_2_SO_4_. ^1^H-NMR (600 MHz, CD_3_OD) *δ* ppm: 5.88 (1H, brs, H-4), 5.79 (1H, d, *J* = 4.5 Hz, H-7), 5.78 (1H, m, H-8), 4.31 (1H, m, H-9), 2.52 (1H, d, *J* = 17.0 Hz, H-2a), 2.16 (1H, d, *J* = 17.0 Hz, H-2b), 1.92 (3 H, d, *J* = 1.3 Hz, H-11), 1.24 (3 H, d, *J* = 6.4 Hz, H-10), 1.04 (3 H, s, H-13), 1.00 (3 H, s, H-12). ^13^C-NMR (150 MHz, CD_3_OD) *δ* ppm: 201.3 (C-3), 167.5 (C-5), 136.9 (C-8), 130.1 (C-7), 127.1 (C-4), 79.9 (C-6), 68.7 (C-9), 50.7 (C-2), 42.4 (C-1), 24.5 (C-13), 23.8 (C-12), 23.5 (C-10), 19.6 (C-11)^15,16^.

Lyratol F (**4**) was obtained as a white powder and developed a dark green coloration upon spraying with 5% vanillin-H_2_SO_4_. ^1^H-NMR (600 MHz, CD_3_OD) *δ* ppm: 5.82 (1H, s, H-8), 4.22 (1H, m, H-3), 2.22 (1H, m, H-2a), 2.19 (3 H, s, H-10), 1.93 (1H, m, H-4a), 1.41 (1H, m, H-2b), 1.38 (3 H, s, H-12), 1.38 (3 H, s, H-13), 1.33 (1H, m, H-4b), 1.15 (3 H, s, H-11). ^13^C-NMR (150 MHz, CD_3_OD) *δ* ppm: 211.6 (C-9), 200.9 (C-7), 119.9 (C-6), 101.1 (C-8), 72.4 (C-5), 64.4 (C-3), 32.2 (C-11), 30.7 (C-13), 29.3 (C-12), 26.5 (C-10)^17^.

(-)-Loliolide (**5**) was was obtained as a white powder and developed a yellow coloration upon spraying with 5% vanillin-H_2_SO_4_. ^1^H-NMR (600 MHz, CDCl_3_) *δ* ppm: 5.68 (1H, s, H-3), 4.32 (1H, m, H-6), 2.46 (1H, dt, *J* = 14.1 and 2.5 Hz, H-5a), 1.98 (1H, dt, *J* = 14.5 and 2.5 Hz, H-7a), 1.78 (3 H, s, H-10), 1.76 (1H, m, H-5b), 1.52 (1H, dd, *J* = 14.5 and 3.7 Hz, H-7b), 1.46 (3 H, s, H-8), 1.27 (3 H, s, H-9). ^13^C-NMR (150 MHz, CDCl_3_) *δ* ppm: 182.7 (C-3a), 172.1 (C-2), 113.0 (C-3), 86.9 (C-7a), 66.9 (C-6), 47.4 (C-5), 45.7 (C-7), 36.1 (C-4), 30.8 (C-8), 27.1 (C-10), 26.6 (C-9)^14^.

Schumannione (**6**) was obtained as a yellow oil and developed a brownish yellow coloration upon spraying with 5% vanillin-H_2_SO_4_. ^1^H-NMR (600 MHz, CD_3_OD) *δ* ppm: 3.68 (1H, dd, *J* = 1.98 Hz, 10.32 Hz, H-5), 1.97 (3 H, s, H-10), 1.79 (3 H, s, H-11), 1.54 (1H, m, H-6b), 1.42 (1H, m, H-6a), 1.36 (2 H, m, H-8), 1.34 (2 H, m, H-7), 0.93 (3 H, m, H-9). ^13^C-NMR (150 MHz, CD_3_OD) *δ* ppm: 174.9 (C-1), 159.9 (C-3), 126.4 (C-2), 109.4 (C-4), 73.4 (C-5), 31.2 (C-6), 29.6 (C-7), 23.7 (C-8), 14.4 (C-9), 11.4 (C-10), 8.3 (C-11)^18^.

Thero-2,3-bis-(4-hydroxy-3-methoxypheyl)-3-methoxy-propanol (**8**) was obtained as a yellow oil and developed a purple coloration upon spraying with 5% vanillin-H_2_SO_4_. ^1^H-NMR (600 MHz, CD_3_OD) *δ* ppm: 6.71 (1H, d, *J* = 8.0 Hz, H-5’), 6.68 (1H, d, *J* = 8.0 Hz, H-5), 6.63 (1H, d, *J* = 1.6 Hz, H-2’), 6.62 (1H, dd, *J* = 1.6 Hz, 8.0 Hz, H-6’), 6.58 (1H, dd, *J* = 1.6 Hz, 8.0 Hz, H-6), 6.48 (1H, d, *J* = 1.6 Hz, H-2), 4.47 (1H, d, *J* = 5.5 Hz, H-7), 3.75, 3.65, 3.15 (each 3 H, s, 3, 3’, 7-OCH_3_), 3.68 (2 H, m, H-9), 2.85 (1H, m, H-8). ^13^C-NMR (150 MHz, CD_3_OD) *δ* ppm: 148.6 (C-3), 148.3 (C-3’), 146.9 (C-4), 146.2 (C-4’), 133.1 (C-1), 132.2 (C-1’), 123.2 (C-6), 121.3 (C-6’), 115.5 (C-2), 115.5 (C-2’), 114.7 (C-5), 111.8 (C-5’), 84.9 (C-7), 64.3 (C-9), 57.0 (C-8), 56.5, 56.3, 56.1 (3, 3’, 7-OCH_3_)^19^.

(7S, 8S)-4-hydroxy-3, 1’, 3’-trimethoxy-4’, 7-epoxy-8, 5’-neolign-9-ol (**9**) was obtained as a brown oil and developed a pink coloration upon spraying with 5% vanillin-H_2_SO_4_. ^1^H-NMR (600 MHz, CD_3_OD) *δ* ppm: 6.64 (1H, d, *J* = 8.0 Hz, H-5), 6.56 (1H, dd, *J* = 1.5 Hz, 8.0 Hz, H-6), 6.52 (1H, d, *J* = 1.5 Hz, H-2), 6.24 (2 H, s, H-2’, 6’), 4.31 (1H, d, *J* = 8.5 Hz, H-7), 4.06 (1H, m, H-9a), 3.92 (1H, m, H-9b), 3.70 (6 H, s, 1’, 3’-OCH_3_), 3.68 (3 H, s, 3-OCH_3_), 2.98 (1H, m, H-8). ^13^C-NMR (150 MHz, CD_3_OD) *δ* ppm: 148.7 (C-1’, 3’), 148.5 (C-3), 146.9 (C-4), 135.1 (C-4’), 132.7 (C-1), 131.8 (C-5’), 121.5 (C-6), 115.5 (C-5), 112.4 (C-2), 107.8 (C-2’, 6’), 87.2 (C-7), 64.8 (C-9), 56.8 (3-OCH_3_), 56.7 (C-8), 56.5 (3’-OCH_3_), 56.3 (1’-OCH_3_)^20^.

1H-indole-3-hydroxyacetyl ZN-21 (**10**) was obtained as a yellow oil and developed a yellow coloration upon spraying with 5% vanillin-H_2_SO_4_. ^1^H-NMR (600 MHz, CD_3_OD) *δ* ppm: 8.23 (1H, d, *J* = 7.9 Hz, H-4), 8.20 (1H, s, H-2), 7.46 (1H, d, *J* = 7.6 Hz, H-7), 7.23 (2 H, m, H-5, H-6), 4.73 (2 H, s, H-9). ^13^C-NMR (150 MHz, CD_3_OD) *δ* ppm: 196.0 (C-8), 138.2 (C-7a), 134.0 (C-2), 126.9 (C-3a), 124.4 (C-6), 123.3 (C-5), 122.6 (C-4), 114.9 (C-3), 112.9 (C-7), 66.3 (C-9)^21,22^.

Methyl hematinate (**11**) was obtained as a white powder and developed a pale purple coloration upon spraying with 5% vanillin-H_2_SO_4_. ^1^H-NMR (600 MHz, CDCl_3_) *δ* ppm: 7.52 (1H, brs, H-1), 3.68 (3H, s, 3’-OCH_3_), 2.70 (2 H, m, H-1’), 2.63 (2 H, m, H-2’), 2.01 (3 H, s, 4-CH_3_). ^13^C-NMR (150 MHz, CDCl_3_) *δ* ppm: 172.6 (C-3’), 171.4 (C-5), 171.2 (C-2), 139.9 (C-3), 139.9 (C-4), 52.0 (3’-OCH_3_), 31.8 (C-2’), 19.5 (C-1’), 8.8 (4-CH_3_)^23^.

Thymine (**12**) was obtained as a white powder and developed a light brown coloration upon spraying with 5% vanillin-H_2_SO_4_. ^1^H-NMR (600 MHz, CD_3_OD) *δ* ppm: 7.22 (1H, s, H-6), 1.84 (3 H, s, H-7). ^13^C-NMR (150 MHz, CD_3_OD) *δ* ppm: 167.5 (C-4), 153.7 (C-2), 139.2 (C-6), 110.4 (C-1), 12.1 (C-7)^24^.

Hydroxy-isobenzofuran-1 (3 H)-oneZN-24 (**13**) was obtained as a yellow oil and developed a bluish-purple coloration upon spraying with 5% vanillin-H_2_SO_4_. ^1^H-NMR (600 MHz, CD_3_OD) *δ* ppm: 5.29 (2 H, s, H-3), 7.07 (1H, d, *J* = 8.04 Hz, H-5), 7.37 (1H, d, *J* = 7.63 Hz, H-6), 7.29 (1H, d, *J* = 7.43 Hz, H-7). ^13^C-NMR (150 MHz, CD_3_OD) *δ* ppm: 174.1 (C-1), 155.0 (C-4), 135.1 (C-3a), 131.7 (C-6), 128.0 (C-7a), 121.6 (C-7), 116.1 (C-5), 69.8 (C-3)^25^.

Pyrocatechol (**14**) was obtained as a white amorphous powder and developed a light blue coloration upon spraying with 5% vanillin-H_2_SO_4_. ^1^H-NMR (600 MHz, CD_3_OD) *δ* ppm: 7.83 (2 H, d, *J* = 7.5 Hz, H-3, 4), 6.74 (2 H, d, *J* = 7.5 Hz, H-2, 5). ^13^C-NMR (150 MHz, CD_3_OD) *δ* ppm: 161.5 (C-1, 6), 115.5 (C-2, 5), 132.5 (C-3, 4)^26^.

Kirenol (**15**) was obtained as white needle-shaped crystals and developed a pale purple coloration upon spraying with 5% vanillin-H_2_SO_4_. ^1^H-NMR (600 MHz, CD_3_OD) *δ* ppm: 5.18 (1H, s, H-14), 3.77 (1H, m, H-2), 3.69 (1H, m, H-15), 3.66 (1H, m, H-16a), 3.56 (1H, dd, *J* = 2.2 Hz, 9.1 Hz, H-16b), 3.46 (1H, m, H-19a), 3.34 (1H, d, *J* = 8.0 Hz, H-19b), 2.28 (1H, m, H-6a), 2.18 (1H, m, H-6b), 2.03 (1H, dd, *J* = 5.2 Hz, 12.8 Hz, H-11a), 2.00 (2 H, m, H-7), 1.81 (1H, t, *J* = 8.6 Hz, H-3a), 1.72 (1H, m, H-3b), 1.57 (2 H, m, H-1), 1.30 (1H, dd, *J* = 4.4 Hz, 12.5 Hz, H-5), 1.21 (1H, dd, *J* = 2.0 Hz, 13.2 Hz, H-9), 1.03 (1H, d, *J* = 11.9 Hz, H-11b), 1.01 (3 H, s, 18-H), 0.93 (1H, m, H-12a), 0.88 (1H, m, H-12b), 0.84 (3 H, s, 17-H), 0.82 (3 H, s, 20-H). ^13^C-NMR (150 MHz, CD_3_OD) *δ* ppm: 139.3 (C-8), 130.1 (C-14), 77.5 (C-15), 65.7 (C-16), 65.2 (C-19), 64.3 (C-2), 56.5 (C-5), 52.5 (C-9), 49.4 (C-1), 45.1 (C-3), 41.4 (C-10), 40.6 (C-4), 38.5 (C-13), 37.4 (C-7), 33.2 (C-12), 28.0 (C-18), 23.3 (C-17), 23.0 (C-6), 19.7 (C-11), 17.2 (C-20)^27^.

8, 3’-dihydroxy-3, 7, 4’-trimethoxy-6-O-β-D-glucopyranosyl (**16**) was obtained as a yellow powder and developed a yellow coloration upon spraying with 5% vanillin-H_2_SO_4_. ^1^H-NMR (600 MHz, CD_3_OD) *δ* ppm: 7.67 (1H, d, *J* = 8.5 Hz, H-6’), 7.63 (1H, s, H-2’), 7.07 (1H, d, *J* = 8.5 Hz, H-5’), 6.91 (1H, s, H-5), 5.12 (1H, d, *J* = 7.4 Hz, H-1’’), 3.95 (3 H, s, 4’-OCH_3_), 3.93 (1H, s, H-6’’b), 3.89 (3 H, s, 7-OCH_3_), 3.82 (3 H, s, 3-OCH_3_), 3.71 (1H, m, H-5’’), 3.58 (1H, m, H-6’’a), 3.57 (1H, m, H-3’’), 3.52 (1H, m, H-2’’), 3.42 (1H, t, *J* = 9.2 Hz, H-4’’). ^13^C-NMR (150 MHz, CD_3_OD) *δ* ppm: 180.4 (C-4), 158.4 (C-2), 157.9 (C-6), 153.8 (C-8), 153.4 (C-9), 151.9 (C-4’), 147.6 (C-3’), 139.9 (C-3), 133.9 (C-7), 124.1 (C-1’), 122.4 (C-6’), 116.3 (C-2’), 112.3 (C-5’), 108.2 (C-10), 101.9 (C-1’’), 95.5 (C-5), 78.5 (C-5’’), 78.0 (C-3’’), 74.8 (C-2’’), 71.3 (C-4’’), 62.5 (C-6’’), 61.5 (7-OCH_3_), 60.6 (3-OCH_3_), 56.4 (4’-OCH_3_)^28^.

Quercetin 3-methyl ether (**17**) was obtained as a pale yellow powder and developed a yellow coloration upon spraying with 5% vanillin-H_2_SO_4_. ^1^H-NMR (600 MHz, CD_3_OD) *δ* ppm: 7.61 (1H, d, *J* = 2. 0 Hz, H-2’), 7.53 (1H, dd, *J* = 2.0 Hz, 8.8 Hz, H-6’), 6.89 (1H, d, *J* = 8.8 Hz, H-5’), 6.36 (1H, s, H-8), 6.17 (1H, d, *J* = 1.8 Hz, H-6), 3.78 (3 H, s, 3-OCH_3_). ^13^C-NMR (150 MHz, CD_3_OD) *δ* ppm: 179.8 (C-4), 167.7 (C-7), 162.9 (C-5), 158.5 (C-9), 157.8 (C-2), 150.1 (C-4’), 146.5 (C-3’), 139.4 (C-3), 122.3 (C-6’), 122.9 (C-1’), 116.4 (C-5’), 116.3 (C-2’), 105.3 (C-10), 100.2 (C-6), 95.1 (C-8), 60.5 (3-OCH_3_)^29^.

p-Hydroxyphenethyl trans-ferulate (**18**) was obtained as a yellow oil and developed a pale purple coloration upon spraying with 5% vanillin-H_2_SO_4_. ^1^H-NMR (600 MHz, CD_3_OD) *δ* ppm: 7.40 (1H, d, *J* = 15.7 Hz, H-7), 7.08 (1H, d, *J* = 1.6 Hz, H-2), 7.02 (2H, d, *J* = 8.4 Hz, H-2’, 6’), 6.98 (1H, dd, *J* = 8.3 Hz, 1.6 Hz, H-6), 6.74 (1H, d, *J* = 8.1 Hz, H-5), 6.68 (2 H, d, *J* = 8.4 Hz, H-3’, 5’), 6.36 (1H, d, *J* = 15.7 Hz, H-8), 3.84 (3 H, s, OCH_3_), 3.43 (2 H, t, *J* = 7.4 Hz, H-1’’), 2.72 (2 H, t, *J* = 7.4 Hz, H-2’’). ^13^C-NMR (150 MHz, CD_3_OD) *δ* ppm: 169.2 (C-9), 156.9 (C-4’), 150.2 (C-4), 149.4 (C-3), 142.1 (C-7), 131.3 (C-1’), 130.7 (C-2’, 6’), 128.1 (C-1), 123.3 (C-6), 118.6 (C-8), 116.5 (C-5), 116.3 (C-3’, 5’), 111.5 (C-2), 56.4 (3-OCH_3_), 42.5 (C-1’’), 35.8 (C-2’’)^30^.

2-Methoxy-4-(2-propenyl)phenyl β-D-glucopyranoside (**19**) was obtained as a white powder and developed a pale yellow coloration upon spraying with 5% vanillin-H_2_SO_4_. ^1^H-NMR (600 MHz, CD_3_OD) *δ* ppm: 7.09 (1H, d, *J* = 8.2 Hz, H-6), 6.82 (1H, d, *J* = 1.5 Hz, H-3), 6.73 (1H, dd, *J* = 1.5 Hz, 8.2 Hz, H-5), 5.94 (1H, m, H-8), 5.06 (1H, d, *J* = 17.1 Hz, H-9b), 5.03 (1H, d, *J* = 10.5 Hz, H-9a), 4.84 (1H, m, H-1’), 3.87 (1H, d, *J* = 11.8 Hz, H-6’a), 3.84 (3 H, s, 2-OCH_3_), 3.69 (1H, m, H-6’b), 3.32–3.48 (6 H, m, H-7, 2’, 3’, 4’, 5’). ^13^C-NMR (150 MHz, CD_3_OD) *δ* ppm: 150.8 (C-2), 146.3 (C-1), 139.0 (C-8), 136.5 (C-4), 122.1 (C-5), 118.3 (C-6), 115.9 (C-9), 114.1 (C-3), 103.1 (C-1’), 78.2 (C-3’), 77.8 (C-5’), 74.9 (C-2’), 71.4 (C-4’), 62.5 (C-6’), 56.7 (2-OCH_3_), 40.8 (C-7)^31^.

4-Allyl-2, 6-dimethoxyphenyl glucoside (**20**) obtained as a white powder and developed a blue coloration upon spraying with 5% vanillin-H_2_SO_4_. ^1^H-NMR (600 MHz, CD_3_OD) *δ* ppm: 6.53 (2H, s, H-2, 6), 5.95 (1H, m, H-8), 5.10 (1H, d, *J* = 17.0 Hz, H-9b), 5.05 (1H, d, *J* = 10.0 Hz, H-9a), 4.81 (1H, d, *J* = 7.6 Hz, H-1’), 3.82 (6 H, s, 3,5-OCH_3_), 3.34 (2 H, d, *J* = 6.8 Hz, H-7). ^13^C-NMR (150 MHz, CD_3_OD) *δ* ppm: 154.2 (C-3), 154.2 (C-5), 138.7 (C-8), 138.4 (C-4), 134.6 (C-1), 116.2 (C-9), 107.5 (C-5), 107.5 (C-3), 105.6 (C-1’), 78.3 (C-3’), 77.8 (C-5’), 75.7 (C-2’), 71.3 (C-4’), 62.6 (C-6’), 57.0 (3-OCH_3_), 57.0 (5-OCH_3_), 41.4 (C-7)^32^.

### Cytotoxicities of isolated compounds 1–20

The twenty isolated compounds from Siegesbeckiae Herba were evaluated for their cytotoxicities against PANC-1 cells using the CCK-8 assay (Table 2). Compounds **1**–**15** and **18**–**20** were essentially inactive, each exhibiting IC_50_ values > 100 µg/mL. In contrast, the two flavonoids **16** and **17** showed measurable cytotoxicity. Compound **16** was the most potent, with an IC_50_ of 4.48 ± 0.74 µg/mL, a level comparable to that of the positive control gemcitabine (IC_50_ = 2.15 ± 0.17 µg/mL) under identical conditions. Compound **17** displayed only moderate inhibition, giving an IC_50_ of 52.04 ± 5.24 µg/mL.

**Table 2:** Cytotoxicities of compounds 1–20 from Siegesbeckiae Herba against PANC−1 cells.

Compound	1–15	16	17	18–20	Gemcitabine^1^
**IC**_**50**_ **± SD (µg/mL)**	> 100	4.48 ± 0.74	52.04 ± 5.24	> 100	2.15 ± 0.17

^1^ Positive control.

### Target identification and network construction of Siegesbeckiae Herba constituents in pancreatic cancer

**Fig. 3:**
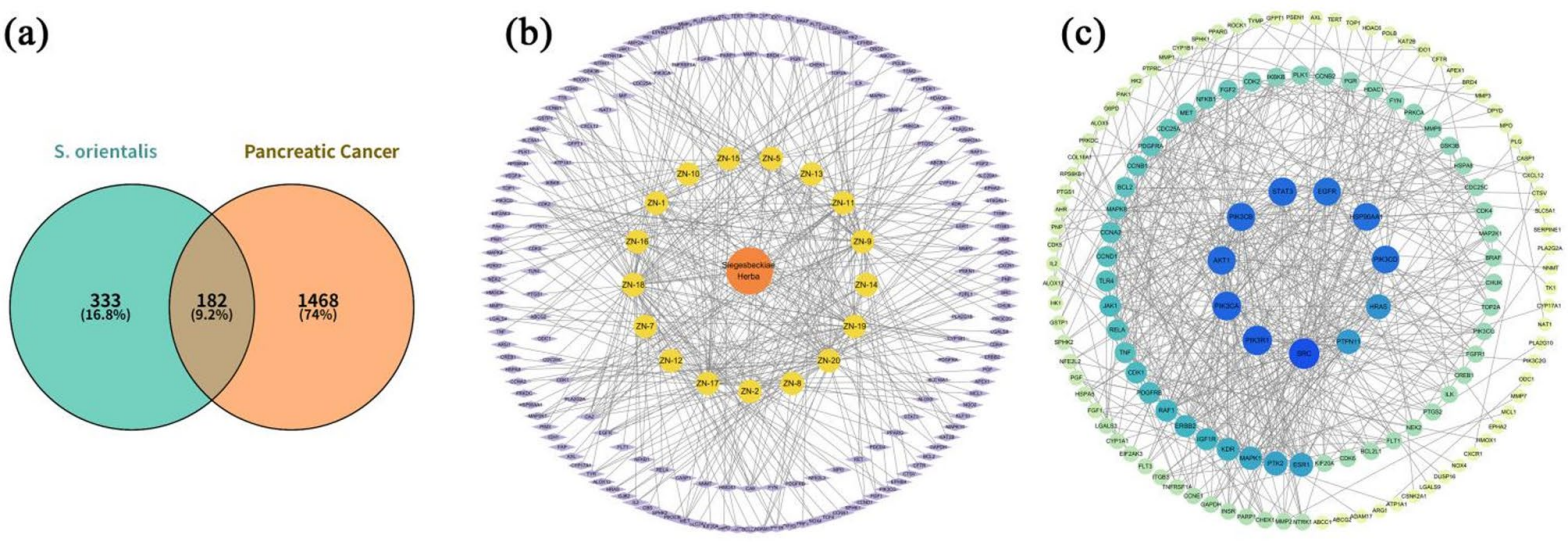
Network pharmacology analysis. (**a**) Venn diagram; (**b**) Target network illustrating anti-pancreatic cancer effects of chemical constituents from Siegesbeckiae Herba; Orange circle represents total chemical constituents, yellow circle represents monomeric compounds, and purple polygons represent pancreatic cancer-related targets; (**c**) PPI network analysis.

To investigate the mechanism of anti-pancreatic cancer effects of Siegesbeckiae Herba, network pharmacology was employed for target prediction and pathway analysis. A total of 515 potential targets for Siegesbeckiae Herba constituents were predicted using the STP and SEA databases. Simultaneously, 1650 pancreatic cancer associated targets were identified using GeneCards, OMIM, and TTD databases. After calibration and deduplication via the UniProt database, 182 intersection targets were identified using Venny software (Fig. 3a). The intersection targets were imported into Cytoscape 3.10.3 software to construct a component-target network, which included 200 nodes and 390 edges, with an average node degree of 3.90 (Fig. 3b). Notably, compounds **17**–**19** displayed interactions with multiple targets, indicating high participation within the network. Compound **17**, which exhibited cytotoxicity against PANC−1 cells, was linked to 55 targets, including key targets such as EGFR, AKT1, CDK1, CDK2, MMP9, PIK3CG, and TERT, closely associated with tumour cell proliferation, migration, and apoptosis. Although Compound **16** exhibited fewer interactions (12 targets), it demonstrated the most significant cytotoxicity against PANC−1, and its targets, including TNF, PTGS2, ALOX5, CYP1B1, and ABCG2, are closely involved in inflammatory and drug resistance.

### PPI network analysis

The 182 overlapping targets were uploaded to the STRING database to construct a PPI network, comprising 144 nodes and 533 edges, with an average node degree of 7.79 (Fig. 3c). Node colour and size indicated node degree, with darker and larger nodes corresponding to higher connectivity. Using Cytoscape 3.10.3 software and the cytoHubba plugin, network topology analysis identified the top 10 core targets (SRC, PIK3R1, PIK3CA, AKT1, STAT3, PIK3CB, HSP90AA1, PIK3CD, EGFR, and HRAS) based on Degree scores. These genes play crucial roles in pancreatic cancer-related biological processes, including cell proliferation, migration, inflammation, signal transduction, and tumour immune response, suggesting their central regulatory roles in mediating the anti-pancreatic cancer effects of Siegesbeckiae Herba.

### GO functional and KEGG pathway enrichment analysis

**Fig. 4:**
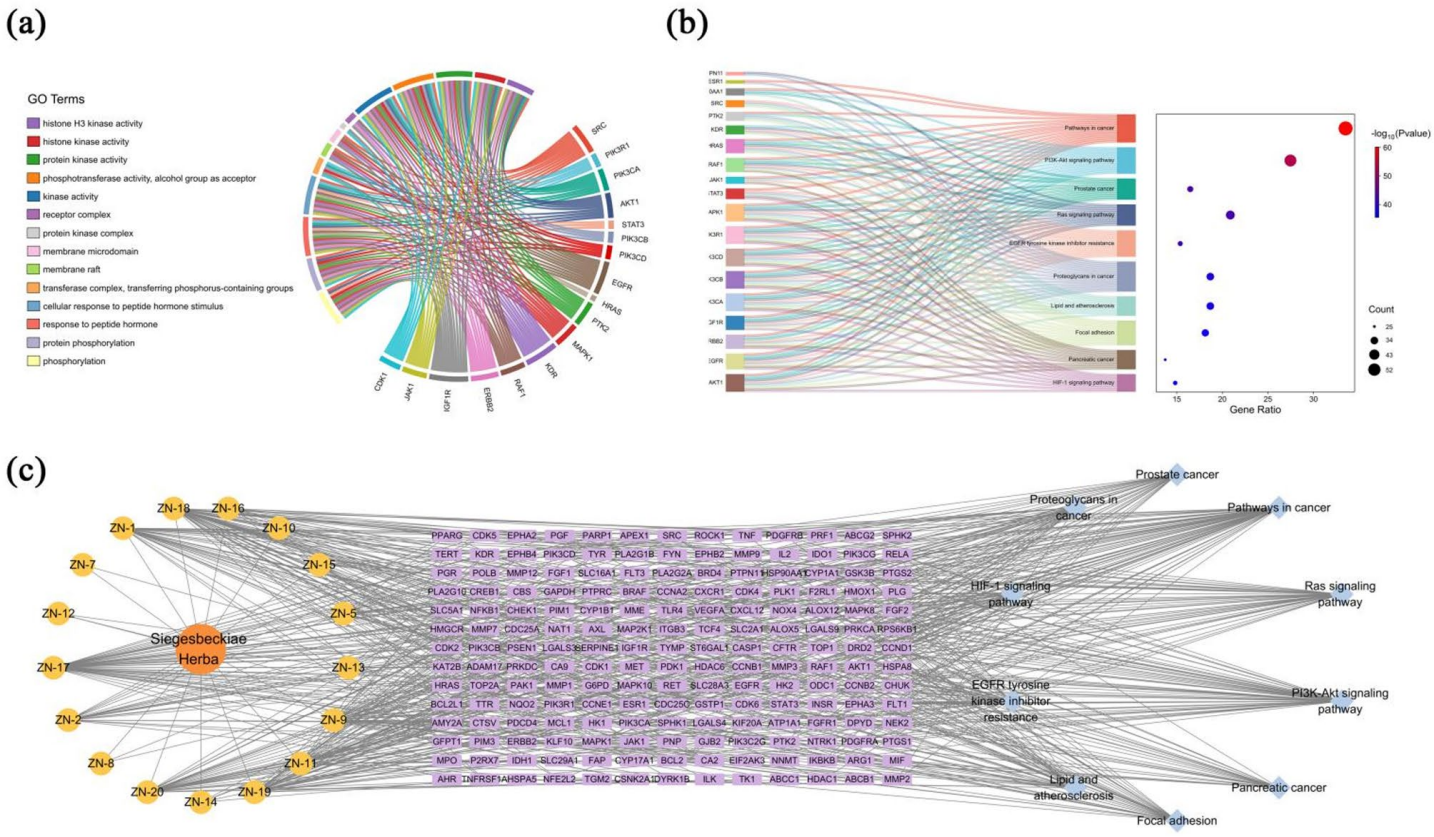
(**a**) GO functional enrichment analysis; (**b**) KEGG pathway analysis; (**c**) Compound-Target-Pathway Network.

To further elucidate the functional process underlying the anti-pancreatic cancer effects of Siegesbeckiae Herba, GO functional enrichment analysis was performed on the 182 overlapping targets using the Metascape platform. This analysis encompassed three primary categories of Biological Process (BP), Molecular Function (MF), and Cellular Component (CC) (Fig. 4a, Table S1). In the BP category, 2003 terms were identified, with significant enrichment in processes such as cellular response to nitrogen compound, phosphorylation, positive regulation of cell migration, and response to peptide hormone stimulus, closely associated with tumour cell signalling, motility, and adaptation to the microenvironment. In the CC category, 103 terms were identified, notably enriched in membrane raft, protein kinase complex, membrane microdomain, and cell-substrate junction, suggesting crucial roles in transmembrane signalling and response to the microenvironment. In the MF category, 256 terms were identified, primarily involving kinase activity such as protein kinase activity, protein tyrosine kinase activity, and histone kinase activity, highlighting the importance of phosphorylation and associated enzymes in dysregulated signalling in cancer cells.

KEGG pathway enrichment analysis was conducted on the 182 overlapping targets using Metascape (Fig. 4b, Table S2). The analysis identified significant enrichment 84 pathways, with pathways in cancer being the most significantly enriched pathway (*p*-value = 6.98E-61), involving 61 genes. This composite pathway integrated multiple oncogenic mechanisms and encompassed several classical tumour related signalling pathways, inclding PI3K-AktPI3K-Akt signaling pathway, Ras signaling pathway, HIF-1 signaling pathway, and EGFR tyrosine kinase inhibitor resistance. These classical tumour related pathways were also significantly enriched individually, playing essential roles in regulating tumour cell proliferation, migration, apoptosis, and drug resistance. Additionally, pancreatic cancer, prostate cancer, proteoglycans in cancer, focal adhesion, and lipid and atherosclerosis pathways, closely associated with tumorigenesis and tumour microenvironment regulation, were also significantly enriched, suggesting that Siegesbeckiae Herba chemical constituents may exert anti-pancreatic cancer effects through multiple pathways (Fig. 4c).

### Molecular docking analysis

**Fig. 5:**
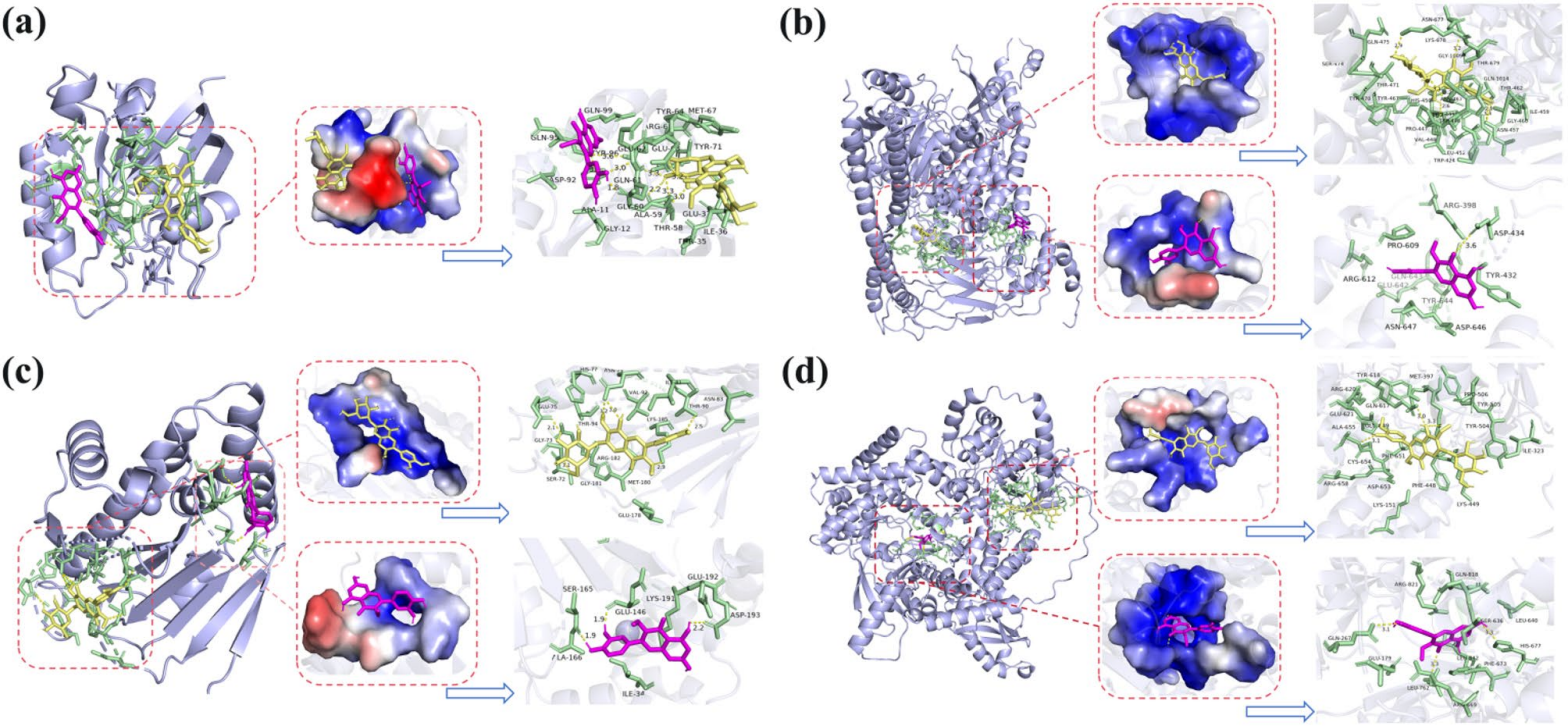
Molecular docking poses of compounds 16 and 17 with key target proteins. Compound 16 is shown in yellow sticks and compound **17** in deep pink sticks; hydrogen bonds are depicted as yellow dashed lines, and interacting amino acid residues are rendered as green sticks and labelled. (**a**) HRAS; (**b**) PIK3CA; (**c**) HSP90AA1; (**d**) PIK3CB.

Molecular docking was conducted to evaluate the interactions between compounds **16**, **17**, and the the top 10 core targets identified from the PPI network analysis (Table 3; Fig. 5). Docking results indicated that both flavonoids exhibited strong binding affinities (<-6.6 kcal/mol) toward all key targets, suggesting stable ligand-target interactions. Particularly, compound **16**, with stronger cytotoxicity, exhibited more pronounced binding affinities (<-7.0 kcal/mol) compared to compound 17. For instance, compound **16** showed strong interactions with HRAS (− 8.76 kcal/mol), PIK3CB (− 8.46 kcal/mol), PIK3CA (− 7.96 kcal/mol), and HSP90AA1 (− 7.83 kcal/mol). For HRAS, both compounds bound to the same active pocket, indicating potentially shared mechanistic pathways. In contrast, for the other three proteins (PIK3CB, PIK3CA, and HSP90AA1), compounds **16** and **17** occupied distinct binding pockets, suggesting differential mechanistic interactions.

**Table 3:** Results of molecular Docking of the top 10 hub genes of pancreatic cancer with compounds 16, 17.

Gene	Uniprot ID	Protein	Compound 16	Compound 17
			Affinity (kcal/mol))	
SRC	P12931	Proto-oncogene tyrosine-protein kinase Src	-7.14	-7.22
PIK3R1	P27986	Phosphatidylinositol 3-kinase regulatory subunit alpha	-7.46	-6.82
PIK3CA	P42336	Phosphatidylinositol 4,5-bisphosphate 3-kinase catalytic subunit alpha isoform	-7.96	-7.03
AKT1	P31749	RAC-alpha serine/threonine-protein kinase	-7.31	-7.35
STAT3	P40763	Signal transducer and activator of transcription 3	-7.26	-7.27
PIK3CB	P42338	Phosphatidylinositol 4,5-bisphosphate 3-kinase catalytic subunit beta isoform	-8.46	-7.67
HSP90AA1	P07900	Heat shock protein HSP 90-alpha	-7.83	-7.57
PIK3CD	O00329	Phosphatidylinositol 4,5-bisphosphate 3-kinase catalytic subunit delta isoform	-7.87	-7.12
EGFR	P00533	Epidermal growth factor receptor	-7.22	-6.63
HRAS	P01112	GTPase HRas	-8.76	-8.08

## Discussion

In this study, twenty compounds were isolated and identified from Siegesbeckiae Herba, among which two flavonoids (compounds **16** and **17**) exhibited cytotoxicity against PANC-1 pancreatic cancer cells. Notably, compound **16** demonstrated potent cytotoxicity comparable to gemcitabine, a first-line chemotherapeutic drug, highlighting its potential as an anti-pancreatic cancer candidate. Flavonoids derived from TCM have increasingly gained attention for their anti-pancreatic cancer properties, primarily through modulation of multiple pathways involved in tumour growth and survival^33^. Given the limited yield of compounds **16** and **17**, network pharmacology and molecular docking were employed to predict their potential anti-cancer mechanisms.

Compound **17** (quercetin-3-methyl ether) interacted with multiple key targets related to pancreatic cancer, including AKT1, EGFR, TNF, CASP3, and SRC, and occupies a central position within the PPI network, indicating the potential regulatory interaction. Research showed that over 90% of pancreatic ductal adenocarcinoma (PDAC) cases harboured KRAS mutations, which activated PI3K/AKT signalling pathways, key drivers of tumour proliferation, drug resistance and metastasis^34^. Although compound **17** was not directly predicted to target KRAS, its interaction with downstream effectors (AKT1), upstream activators (EGFR), and modulators (SRC) within the KRAS signaling network suggests its potential interference with this oncogenic axis at multiple levels^35–37^. Furthermore, existing research indicated compound **17** suppressed tumur stemness, proliferation, migration, and epithelial-mesenchymal transition (EMT) in breast cancer by inhibiting the PI3K/Akt/mTOR pathway and stemness-related genes, such as SRY-box2 and Nanog^38^. Although direct evidence in pancreatic cancer is currently lacking, these predicted targets provide a valuable theoretical basis for future pharmacological validation.

In comparison, compound **16** (8, 3’-dihydroxy-3, 7, 4’-trimethoxy-6-O-β-D-glucopyranosyl) had fewer predicted pancreatic cancer-related targets and did not prominently feature in the PPI network. However, it demonstrated strong cytotoxic activity in the CCK-8 assay, suggesting that the current target prediction may not fully capture its mechanism of action. Structural analysis indicated compound **16** as a flavonoid glycoside, whereas compound **17** was a flavonoid, suggesting differences in substituent position and type potentially underlie their divergent biological activities. Previous studies demonstrated that flavonoids exert anti-cancer effects through various pathways, as exemplified by apigenin inducing cell-cycle arrest and apoptosis of the cells, quercetin enhancing chemotherapy sensitivity by inhibiting the AGE/PI3K/AKT/mTOR pathway^39,40^. Mazurakova et al. (2023) reported flavonoids reversing tumour cell resistance by modulating the PI3K/Akt-HIF-1α signalling axis^41^. Although compound **16** was not enriched in the top-ranked predicted pathways identified by KEGG analysis in this study, its predicted targets such as TNF, ALOX5, PTGS2, CYP1B1 were associated with inflammation, lipid metabolism, and drug resistance, suggesting potential anti-tumour effects possibly through non-classical pathways. Further exploration linking structural features to biological functions is warranted.

GO enrichment analysis revealed significant involvement of Siegesbeckiae Herba constituents in lipid rafts and cellular responses to nitrogen compounds. Lipid rafts are critical microdomains for signal transduction, concentrating receptors and kinases involved in regulating cancer cell proliferation, migration, survival, and apoptosis through pathways like PI3K/AKT^42^. Pancreatic cancer cells rely heavily on glutamine as a nitrogen source, regulating cell proliferation, autophagy inhibition, and metabolic reprogramming through the mTORC1 signalling^43^. Moreover, KEGG pathway enrichment indicated anti-pancreatic cancer effects closely linked to the PI3K-Akt/mTOR signaling axis, one of the most frequently activated pathways in cancers, promoting cell survival, proliferation, and resistance to anticancer therapies^44^. The HIF-1 signalling pathway, essential for cellular adaptation to hypoxia via HIF-1α, was also enriched, further supporting a role in tumour microenvironment adaptation and progression in pancreatic cancer^45^. Although some crucial pathways (e.g., MAPK, Apoptosis, ERBB, JAK-STAT) were not top-ranked, core genes within these pathways (AKT1, EGFR, CASP3, TNF, SRC) showed high connectivity in the PPI network, reinforcing their relevance to the anti-cancer mechanisms of Siegesbeckiae Herba constituents.

Collectively, these findings provide valuable guidance for selecting bioactive constituents and outline future directions for mechanistic and pharmacological validation. Future studies should prioritize chemical synthesis and structural optimization of compound **16**, conduct comprehensive mechanistic investigations through in vitro and in vivo models, and undertake thorough safety evaluations to assess clinical potential.

## Methods

### General information

Semi-preparative HPLC analyses were performed on Agilent Technologies 1260 Infinity instruments (Agilent Technologies, CA, USA) equipped with an Agilent C18 column (250 × 9.4 mm, 5 μm). NMR spectra were recorded on a Bruker AV-600 spectrometer (600 MHz for ¹H and 150 MHz for ¹³C, Bruker, Karlsruhe, Germany), and high-resolution electrospray ionization mass spectra (HR-ESI-MS) were acquired using an Agilent 6545 UPLC/Q-TOF mass spectrometer (CA, USA). Absorbance at 450 nm was measured using SpectraMax microplate reader (Shanghai, China). TLC analysis was conducted on silica gel G plates (Qingdao Haiyang Chemical Co., Ltd., Qingdao, China). Column chromatography was performed using silica gel (100–200 mesh, 200–300 mesh, Qingdao Haiyang Chemical Co., Ltd., Qingdao, China), Sephadex LH-20 (General Electric, MA, USA), and MCI gel CHP20 (Mitsubishi Chemical Corporation, Tokyo, Japan). Deuterated solvents (CDCl_3_, CD₃OD, DMSO-*d*_*6*_) were obtained from AnnoRoad Biotech (Beijing, China) and Sigma-Aldrich (Shanghai, China). All the analytical solvents of analytical grade purchased from Tianjin Hengxing Chemical Reagent Manufacturing Co., Ltd. (Tianjin, China) and Tianjin Yongda Chemical Reagent Co., Ltd. (Tianjin, China). DMEM and fetal bovine serum were purchased from Gibicol Inc. (New York, USA). Penicillin and streptomycin were obtained from Solarbio Science & Technology Co., Ltd. (Beijing, China). The CCK-8 kit was supplied by G-clone Biotechnology Co., Ltd. (Beijing, China). Gemcitabine, used as the positive control, was purchased from MedChemExpress (MCE, Monmouth Junction, NJ, USA).

### Plant material

The dried Siegesbeckiae Herba was purchased from Anhui Yicaokang Pharmaceutical Co., Ltd. (Bozhou, Anhui Province, China) and was identified by Dr. Xu, Y.B. (Taizhou University Hospital, Taizhou, China). The voucher specimen (TZZXYY-Xuyubin-2023-02-001) was stored at Taizhou University Hospital.

### Extraction and isolation

The dried Siegesbeckiae Herba (30 kg) was immersed in 75% ethanol at room temperature for 3 days with continuous stirring. The extract was filtered, concentrated, and the extraction procedure was repeated once. The combined concentrated extracts were diluted with water and successively extracted three times each with petroleum ether and ethyl acetate. The resulting petroleum ether, ethyl acetate, and aqueous fractions were separately concentrated under reduced pressure. The ethyl acetate extract was subjected to silica gel (100–200 mesh) column chromatography and eluted with a stepwise gradient of dichloromethane-methanol (100:0, 100:1, 50:1, 30:1, 20:1, v/v). Eluates were monitored by TLC and combined into seven fractions (Fr.1-Fr.7). Fraction Fr.3 was further separated to yield two subfractions (Fr.3 − 1 and Fr.3 − 2), which were combined with Fr.2 and Fr.4 to form Fr.A and Fr.B, respectively.

Further chromatographic purification employing MCI, Sephadex LH-20, and semi-preparative HPLC on the Fr.A fraction resulted in the isolation of compounds ZN-1 (18.2 mg), ZN-2 (4.0 mg), ZN-3 (1.2 mg), ZN-4 (9.2 mg), ZN-5 (7.8 mg), ZN-6 (3.2 mg), ZN-7 (4.2 mg), ZN-8 (1.2 mg), ZN-9 (4.0 mg), ZN-10 (0.9 mg), ZN-11 (2.3 mg), ZN-12 (3.7 mg), ZN-13 (3.8 mg), and ZN-14 (4.8 mg). Similarly, fraction Fr.B yielded compounds ZN-15 (560.0 mg), ZN-16 (4.4 mg), ZN-17 (11.6 mg), ZN-18 (4.5 mg), ZN-19 (4.3 mg), and ZN-20 (4.3 mg). All isolated compounds were stored at 4 °C for subsequent analyses.

### Cytotoxicity assay

The cytotoxicities of the isolated compounds ZN-1 to ZN-20 were assessed against human pancreatic cancer PANC-1 cells using the standard Cell Counting Kit (CCK)-8 assay^46^. PANC-1 cells were obtained from National Collection of Authenticated Cell Cultures (Shanghai, China) and maintained in DMEM medium supplemented with 10% fetal bovine serum, 100 U/mL penicillin, and 0.1 mg/mL streptomycin. Cells were incubated at 37 ℃ in a CO_2_ incubator containing 5% CO_2_. Cells were seeded into 96-well plates at a density of 6 × 10^3^ cells per well and cultured 20–24 h to allow attachment. Subsequently, the culture medium was replaced with fresh medium containing serial dilutions of each test compound and incubated for 48 h. Then, 10 µL of CCK-8 reagent was added to each well, and cells were incubated for 1 h. Absorbance was measured at 450 nm using a microplate reader. The 50% inhibitory concentration (IC_50_) values were calculated using Probit analysis with SPSS software (version 20.0, NY, USA).

### Network pharmacology

Twenty compounds isolated from Siegesbeckiae Herba were submitted to the Swiss Target Prediction (STP, https://www.swisstargetprediction.ch/) database and Similarity Ensemble Approach (SEA, https://sea.bkslab.org/) database to predict targets in *Homo sapiens* mode^47,48^. Through the STP platform, targets with probability greater than 0 were screened as potential targets. Through the SEA platform, targets with an exact structural match to known ligands (MaxTC = 1.0) and targets identified as significant matches by the platform were selected as high-confidence targets. All prediction results were standardised via the UniProt database (https://www.uniprot.org/)^49^.

Pancreatic cancer targets were collected by searching the GeneCards (https://www.genecards.org/), OMIM (https://www.omim.org/), and TTD (https://idrblab.org/ttd/) databases^50–52^. After calibration and deduplication through the UniProt database, the human pancreatic cancer-related targets were obtained. Subsequently, the online tool Venny2.1 (https://bioinfogp.cnb.csic.es/tools/venny/) was utilised to identify the intersection genes between disease targets and predicted compound targets. Cytoscape 3.10.3 software was used to construct and visualize the compound-target interaction network^53^. The intersecting targets were inputted into the STRING database (https://string-db.org/) with the species set to “*Homo sapiens*” and a minimum interaction score of 0.9^54^. The resulting PPI network was imported into Cytoscape software, where network topology analysis was performed using the cytoHubba plugin based on Degree values to identify the top 10 hub genes.

To further elucidate the potential mechanism of action, the intersecting targets were subjected to GO and KEGG pathway enrichment analysis using the Metascape platform (https://metascape.org/)^55–59^. The GO and KEGG enrichment analysis were plotted by https://www.bioinformatics.com.cn, an online platform for data analysis and visualization^60^.

### Molecular docking

To elucidate intermolecular interactions, molecular docking was performed using compounds **16**, **17** and key targets. The 3D structures of compounds **16** and **17** were retrieved from the TCMSP database (https://old.tcmsp-e.com/index.php), and the crystal structures of target proteins were obtained from the UniProt database (https://www.uniprot.org/). Receptor-ligand docking was conducted on the DockThor platform (https://dockthor.lncc.br/)^61^. Docking results were expressed as binding energies (kcal/mol), with negative values indicating spontaneous interactions between receptors and ligands. The docking poses were visualised using PyMOL 2.5 software.

## Conclusions

Twenty compounds, including one new compound, were isolated from Siegesbeckiae Herba. Bioactivity evaluation revealed two flavonoids exhibited significant cytotoxicity against PANC-1 cells. Network pharmacology analysis indicated these compounds act through multiple targets within critical cancer-related pathways, particularly the PI3K-Akt, Ras, and HIF-1 signaling pathways. Molecular docking further differentiated the mechanistic interactions of compounds **16** and **17**, showing that the two ligands bind to distinct active pockets on the same key targets. Compound **16** demonstrated particularly strong binding affinities toward HRAS, PIK3CB, PIK3CA, and HSP90AA1, supporting its significant cytotoxic potential and providing compelling evidence for its therapeutic promise. Future research should focus on the chemical synthesis and optimization of compound **16**, detailed mechanistic validation in vitro and in vivo, and thorough safety assessment for potential clinical translation.

## Supplementary Information

Below is the link to the electronic supplementary material.

Supplementary Material 1

## Data Availability

The datasets used and analysed during the current study available from the corresponding author on reasonable request.
