# Supplementary material for: Identification of cytotoxic constituents from Siegesbeckiae Herba and network pharmacology prediction of their anti-pancreatic cancer mechanisms
Source: Sci Rep. 2025 Sep 26;15:33008. doi: 10.1038/s41598-025-18358-3 (PMC12475171; doi:10.1038/s41598-025-18358-3)
Supplement: Supplementary file 1 — Supplementary Material 1 [file 41598_2025_18358_MOESM1_ESM.docx]

**Tables**

**Table S1 Top 10 GO Enrichment for Anti-Pancreatic Cancer Targets**

| **NO.** | **GO accession** | **Term type** | **Description** | ***p*-Value** | **Gene Number** |
| --- | --- | --- | --- | --- | --- |
| 1 | GO:1901699 | BP | Cellular response to nitrogen compound | 7.75E-55 | 60 |
| 2 | GO:0016310 | BP | Phosphorylation | 4.02E-54 | 53 |
| 3 | GO:0006468 | BP | Protein phosphorylation | 4.18E-52 | 50 |
| 4 | GO:0043434 | BP | Response to peptide hormone | 9.36E-50 | 50 |
| 5 | GO:0071375 | BP | Cellular response to peptide hormone stimulus | 8.92E-47 | 43 |
| 6 | GO:0030335 | BP | Positive regulation of cell migration | 1.65E-46 | 53 |
| 7 | GO:2000147 | BP | Positive regulation of cell motility | 2.16E-45 | 53 |
| 8 | GO:0040017 | BP | Positive regulation of locomotion | 8.08E-45 | 53 |
| 9 | GO:0032870 | BP | Cellular response to hormone stimulus | 6.25E-44 | 50 |
| 10 | GO:0007169 | BP | Cell surface receptor protein tyrosine kinase signaling pathway | 1.33E-42 | 45 |
| 1 | GO:0061695 | CC | Transferase complex, transferring phosphorus-containing groups | 1.61E-17 | 22 |
| 2 | GO:0045121 | CC | Membrane raft | 1.28E-15 | 20 |
| 3 | GO:0098857 | CC | Membrane microdomain | 1.46E-15 | 20 |
| 4 | GO:1902911 | CC | Protein kinase complex | 1.54E-15 | 16 |
| 5 | GO:0043235 | CC | Receptor complex | 2.95E-14 | 24 |
| 6 | GO:1902554 | CC | Serine/threonine protein kinase complex | 1.63E-13 | 14 |
| 7 | GO:0005925 | CC | Focal adhesion | 1.49E-12 | 20 |
| 8 | GO:0031983 | CC | Vesicle lumen | 1.85E-12 | 18 |
| 9 | GO:0030055 | CC | Cell-substrate junction | 2.28E-12 | 20 |
| 10 | GO:0000307 | CC | Cyclin-dependent protein kinase holoenzyme complex | 2.33E-12 | 10 |
| 1 | GO:0016301 | MF | Kinase activity | 1.68E-59 | 66 |
| 2 | GO:0016773 | MF | Phosphotransferase activity, alcohol group as acceptor | 7.19E-59 | 64 |
| 3 | GO:0004672 | MF | Protein kinase activity | 1.67E-53 | 57 |
| 4 | GO:0035173 | MF | Histone kinase activity | 3.79E-46 | 45 |
| 5 | GO:0140996 | MF | Histone H3 kinase activity | 2.40E-43 | 43 |
| 6 | GO:0140993 | MF | Histone modifying activity | 6.71E-43 | 52 |
| 7 | GO:0141003 | MF | Histone H2AX kinase activity | 4.86E-42 | 42 |
| 8 | GO:0035401 | MF | Histone H3Y41 kinase activity | 4.30E-32 | 25 |
| 9 | GO:0140801 | MF | Histone H2AXY142 kinase activity | 4.30E-32 | 25 |
| 10 | GO:0004713 | MF | Protein tyrosine kinase activity | 8.86E-30 | 25 |

**Table S2 Top 10 KEGG Pathway Enrichment Results for Anti-Pancreatic Cancer Targets**

| **NO.** | **ID** | **Term** | ***p*-Value** | **Gene Number** |
| --- | --- | --- | --- | --- |
| 1 | hsa05200 | Pathways in cancer | 6.98E-61 | 61 |
| 2 | hsa04151 | PI3K-Akt signaling pathway | 1.14E-53 | 50 |
| 3 | hsa05215 | Prostate cancer | 1.93E-43 | 30 |
| 4 | hsa04014 | Ras signaling pathway | 4.27E-43 | 38 |
| 5 | hsa01521 | EGFR tyrosine kinase inhibitor resistance | 1.64E-42 | 28 |
| 6 | hsa05205 | Proteoglycans in cancer | 3.15E-39 | 34 |
| 7 | hsa05417 | Lipid and atherosclerosis | 2.47E-38 | 34 |
| 8 | hsa04510 | Focal adhesion | 1.06E-37 | 33 |
| 9 | hsa05212 | Pancreatic cancer | 4.75E-37 | 25 |
| 10 | hsa04066 | HIF-1 signaling pathway | 3.48E-36 | 27 |

**Figures**

**
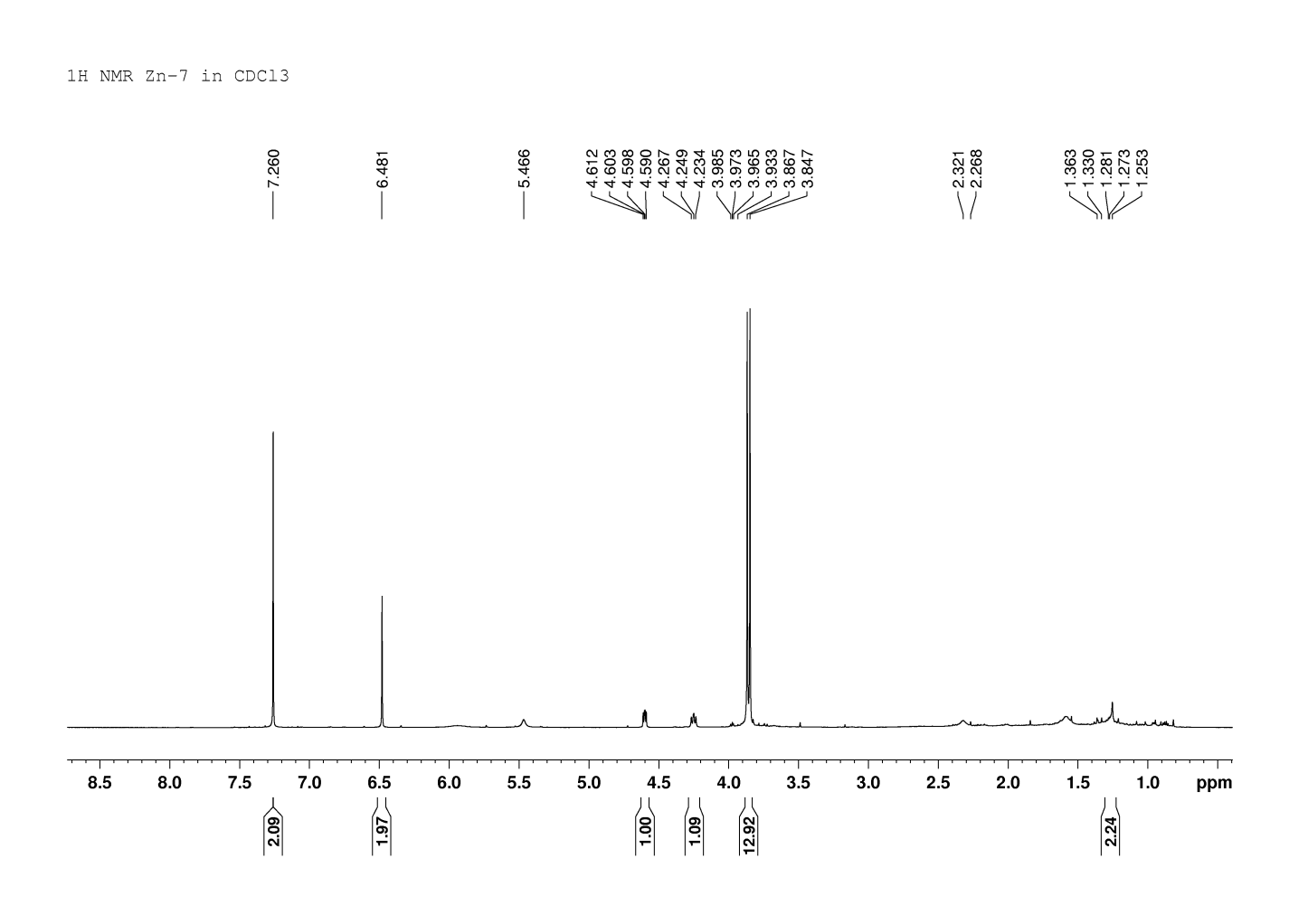
**

**Figure S1.** ^1^H-NMR spectrum of compound **7**.

**

**

**Figure S2.** ^13^C-NMR spectrum of compound **7**.


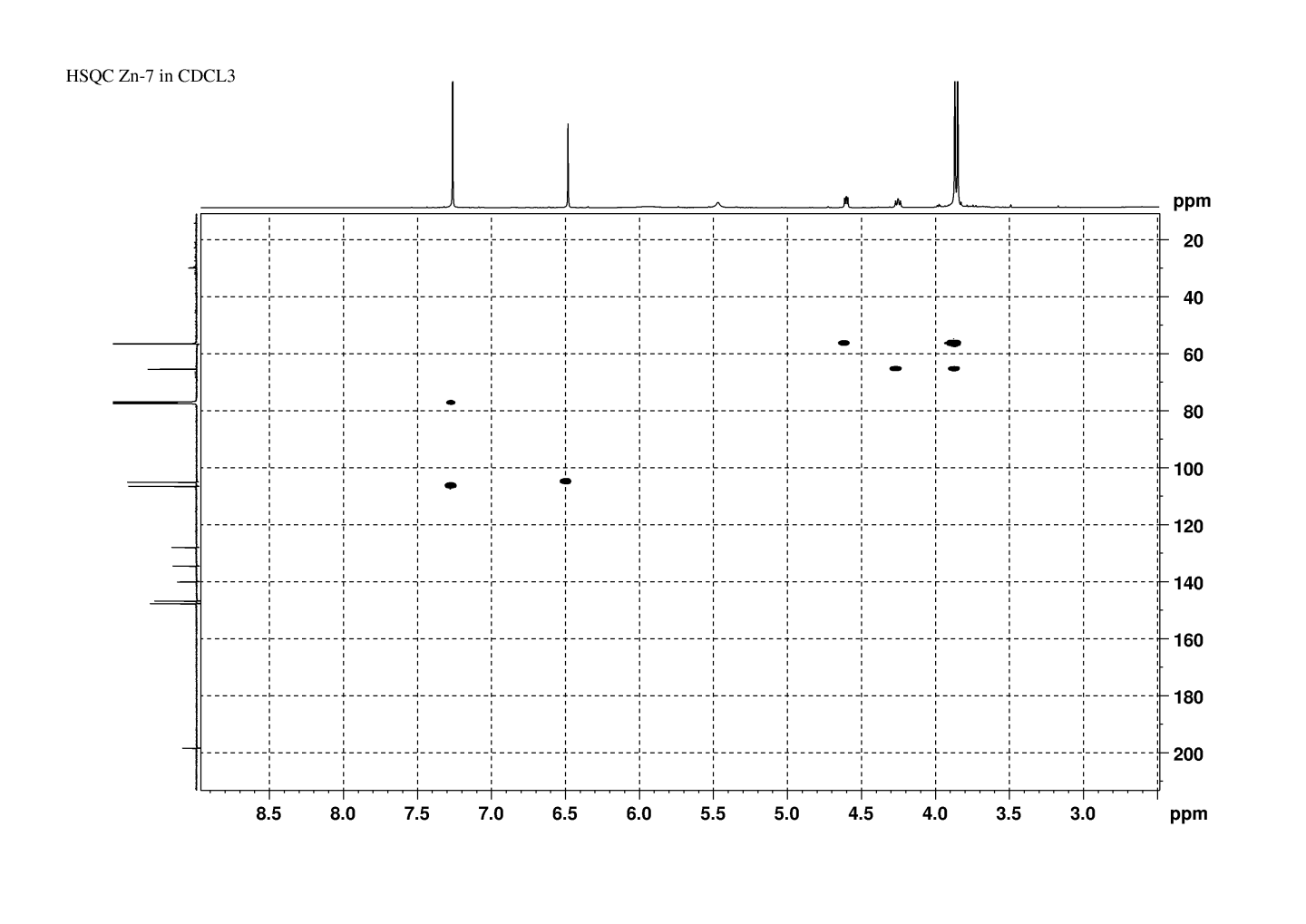


**Figure S3.** HSQC spectrum of compound **7**.


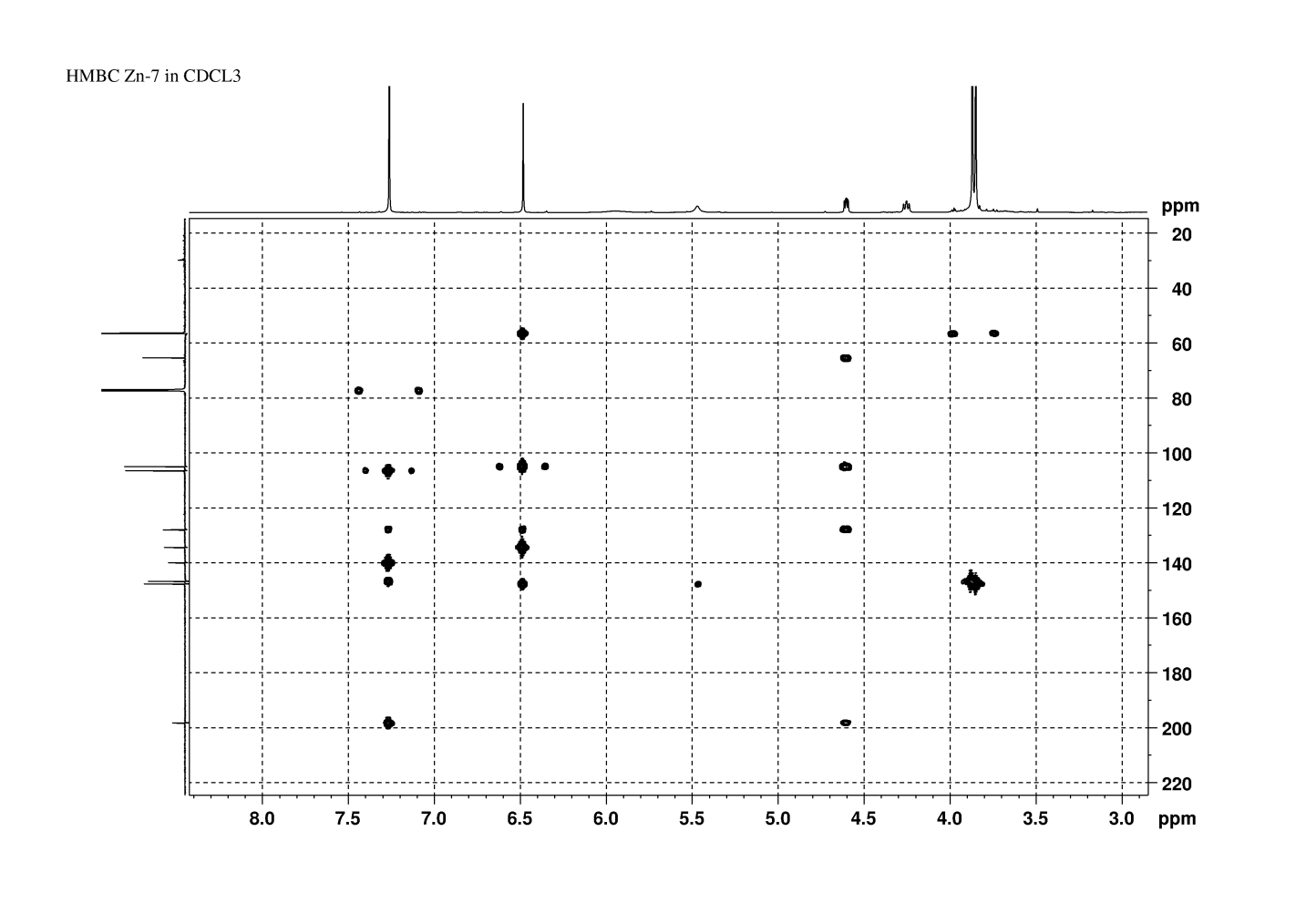


**Figure S4.** HMBC spectrum of compound **7**.


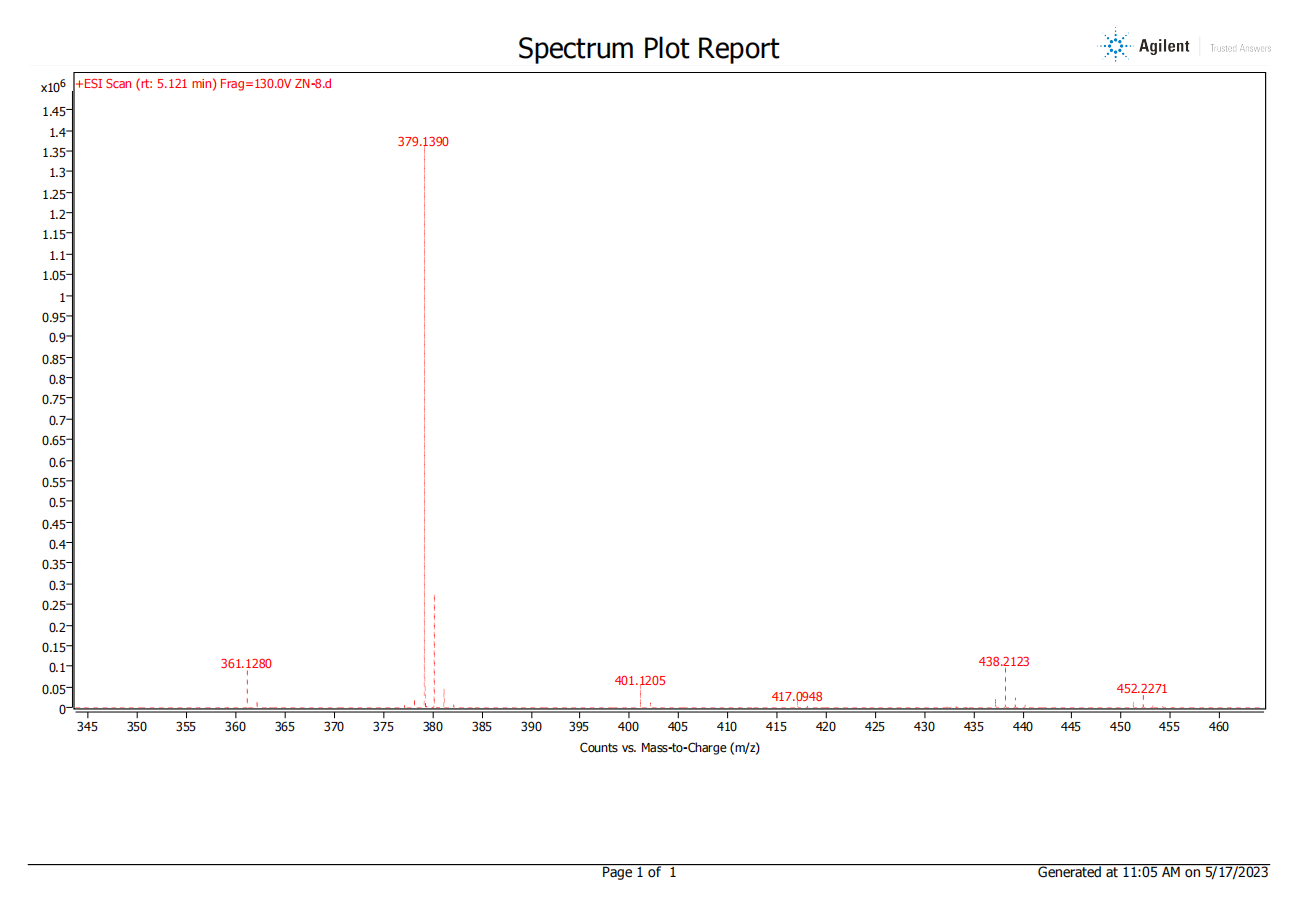


**Figure S5.** ESI-MS of compound **7**.
